# Improvement of ovarian response and oocyte quality of aged female by administration of bone morphogenetic protein-6 in a mouse model

**DOI:** 10.1186/1477-7827-10-117

**Published:** 2012-12-29

**Authors:** Seung S Park, Min J Park, Bo S Joo, Jong K Joo, Jung B Son, Kyu S Lee

**Affiliations:** 1 Department of Obstetrics and Gynecology, Medical Research Institute, Pusan National University School of Medicine, Busan, Korea; 2 Center for Reproductive Medicine, Good Moonhwa Hospital, Busan, Korea

**Keywords:** Female aging, BMP-6, Ovarian function, Oocyte quality, Id-1, VEGF

## Abstract

**Background:**

Advancing female age remains a difficult problem in infertility treatment. Ovarian angiogenesis plays an important role in follicular development and the activation of ovarian angiogenesis has been emerged as a new strategy for the improvement of age-related decline of oocyte quality. BMP-6 affect gonadotropin signals in granulosa cells and it promotes normal fertility by enabling appropriate response to LH and normal oocyte quality. BMP-6 has a potential role in regulation of angiogenesis and regulates the expression of inhibitor of DNA-binding proteins (Ids). Ids involved in the control and timing of follicle selection and granulosa cells differentiation. Especially, Id-1 is well-characterized target of BMP-6 signaling. Therefore, this study investigated whether co-administration of BMP-6 during superovulation process improves ovarian response, oocyte quality and expression of Id-1 and vascular endothelial growth factor (VEGF) in the ovary of aged female using a mouse model.

**Methods:**

Aged C57BL/6 female mice (26–31 weeks old) were superovulated by injection with 0.1 mL of 5 IU equine chorionic gonadotropin (eCG) containing recombinant mouse BMP-6 at various doses (0, 0.01, 0.1, 1, and 10 ng), followed by injection with 5 IU human chorionic gonadotropin (hCG) 48 h later. Then, the mice were immediately paired with an individual male. The aged control group was superovulated without BMP-6. Young mice of 6–9 weeks old were superovulated without BMP-6 as a positive control for superovulation and *in vitro* culture of embryos. Eighteen hours after hCG injection, zygotes were retrieved and cultured for 4 days. Both ovaries of each mouse were provided in the examination of ovarian expression of Id-1 and VEGF by reverse transcriptase-polymerase chain reaction, western blot, and immunohistochemistry.

**Results:**

Administration of 0.1 ng BMP-6 significantly increased the number and blastocyst formation rate of oocytes ovulated and ovarian expression of Id-1 and VEGF compared to aged control mice. These increased levels were comparable to those of young control mice.

**Conclusions:**

This result suggests that BMP-6 during ovulation induction plays an important role in improvement of oocyte quality and ovarian response of aged female, possibly by regulating of ovarian Id-1 and VEGF expression.

## Background

Despite advancing female age becomes an important factor for increasing incidence of infertility, it remains a difficult problem in infertility treatment. A major cause for age-related decline of fertility is the deterioration of oocyte quality and ovarian response [1,2]. However, there is no clinically effective method to improve the oocyte quality in old women.

Ovarian angiogenesis plays an important role in follicular growth and the selection of dominant follicle by allowing the delivery of adequate nutrition, hormonal supply and oxygen from stromal blood vessels to induce oocytes with high quality [3-6]. Tatone *et al*. [7] suggested a possible mechanism that age-related nuclear and cytoplasmic damage may occur as a result of inadequate ovarian angiogenesis in primordial follicles as well as in ovarian stroma vessels. These results suggest that the activation of ovarian angiogenesis could be a new strategy for the improvement of age-related decline of oocyte quality.

Bone morphogenetic proteins (BMPs) are members of the transforming growth factor β (TGF-β) superfamily which plays a critical role in the development of mammalian ovarian follicles [8,9]. Mutations in the *Bmp* genes or their receptors result in impaired female fertility such as decreased granulosa cell proliferation, abnormal oocyte growth, and failure of follicle development [10-13]. The BMP-6 is highly expressed in mammalian oocytes as well as other cell types [14,15]. *In vitro* extensive studies have showed that BMP-6 seems to affect gonadotropin signals in granulosa cells of several mammalian species, although it offered conflicting results [16-21]. *In vivo* study using *BMP-6* null mice also demonstrated that BMP-6 promotes normal fertility by enabling appropriate response to LH and normal oocyte quality [22].

Besides this role, BMP-6 has a potential role in regulation of angiogenesis by affecting endothelial cells (ECs) differentiation, migration, proliferation, and tube formation [23,24]. BMP-6 regulates the expression of inhibitor of DNA-binding proteins (Ids) in the ovine ovarian follicles [25]. Id proteins are negative transcriptional regulators, which lack a basic DNA-binding domain and block subsequent transcriptional activity by forming heterodimer with helix-loop-helix (bHLH) transcription factor [26]. It has been speculated that Ids are involved in the control and timing of follicle selection and granulosa cells differentiation in avian species [27,28]. Mammalian Id superfamily consists of four isoforms (Id-1, Id-2, Id-3, and Id-4) [29]. Especially, Id-1 is a well-characterized target of BMP-6 signaling [23,24] and plays a role in angiogenesis through activation of the vascular endothelial growth factor (VEGF) [30,31].

However, to our knowledge, there have been no studies on the effects of BMP-6 on ovarian response and angiogenesis, and oocyte quality according to the female age. This study examined whether BMP-6 administration during superovulation process affects ovarian response, oocyte quality and expression of Id-1 and VEGF in the ovary of aged female using a mouse model.

## Methods

This study was approved by the Institutional Review Board of Pusan National University Hospital, Korea. All experiments with mice were conducted in accordance with the Guide for the Care and Use of Laboratory Animals of the National Institutes of Health, approved by the Pusan National University Hospital Institutional Animal Care and Use Committee.

### Animals

C57BL/6 inbred female mice were purchased from Korea Experimental Animal Center (Daegu, Korea). The mice were maintained on light(L)-dark(D) cycle with 14/10 h L/D with food and water available ad *libitum*.

### Administration of BMP-6 during superovulation

Aged female mice of 26–31 weeks old were superovulated by intraperitoneal injection with 0.1 mL of 5 IU equine chorionic gonadotropin (eCG) (Sigma, St. Louis, MO, USA) containing various doses (0, 0.01, 0.1, 1, and 10 ng) of recombinant mouse BMP-6 (R&D Systems, Minneapolis, MN, USA), followed by injection of 5 IU of human chorionic gonadotropin (hCG, Sigma) approximately 48 hours later. Then the mice were immediately paired with an individual male that previously tested for fertility. The following morning the mice were inspected, and those with a confirmed vaginal plug were considered mated and fertilized. The aged control group was superovulated without BMP-6. Young mice of 6–9 weeks old were also superovulated without BMP-6 as a positive control for ovarian stimulation and *in vitro* culture of embryos.

### Zygotes collection and *in vitro* culture of embryos

Eighteen hours after hCG injection, female mice with a confirmed vaginal plug were sacrificed by cervical dislocation. Cumulus-enclosed one-cell embryos (zygotes) were retrieved from the oviductal ampulae and denuded by incubation for 1 minute with 0.1% hyaluronidase (Sigma) in Dulbecco's phosphate buffered saline (dPBS; Gibco BRL, Grand Island, NY, USA). Zygotes retrieved from each were individually pooled and washed three times in P1 medium (Irvine Scientific, Santa Ana, CA, USA) with 10% serum substitute supplement (SSS; Irvine Scientific). All the zygotes except for those with cell fragmentation were cultured in 30 μl drops of P1 medium with 10% SSS for the first 2 days, and then blastocyst medium (Irvine Scientific) with 10% SSS for the later 2 days under paraffin-oil at 37°C in a 5% CO_2_ humidity incubator. The media were changed daily as 30 μL-drop culture. When the retrieved one cell embryos developed to 2-cells embryo in the first day of culture, we included the data in this study. However, unless all retrieved one cell embryos developed to 2-cell embryo, we excluded the data of the mouse without developed 2-cell embryos. In this respect, we defined the retrieved one-cell embryo as real zygotes.

### Ovary collection and examination of ovarian Id-1 and VEGF expression

Just after the retrieval of the zygotes, both ovaries of each mouse were collected. For the examination of ovarian expression of Id-1 and VEGF, each ovary was randomly allocated to half for reverse transcriptase-polymerase chain reaction (RT-PCR), half for western blot and a whole for immunohistochemistry (IHC).

### RT-PCR

Total RNA was isolated from frozen tissue using Trizol reagent (Invitrogen, Carlsbad, CA, USA) according to the manufacturer’s recommendation. RNA was eluted in RNase-free water and stored at −80°C until RT-PCR analysis. The cDNA was synthesized from 5 μg of total RNA with AMV Reverse Transcriptase (Promega, Madison, WI, USA) using a random hexamer (Bioneer, Daejeon, Korea) at 42°C for 1 hour followed by inactivate the enzyme at 95°C for 5 minutes. Template cDNA was subjected to PCR amplification using gene-specific sense and antisense primers. PCR conditions were denaturation at 95°C for 30 seconds, specific annealing temperature for 30 seconds, and extension at 72°C for 30 seconds in a thermal cycle. The primers of each gene are as follows: 5’-CTGCTCTACGAC ATGAACGGCTG -3’ (sense)  and 5’-CGACACAAGATGCGATCGTC -3’ (antisense) for Id-1 (32  cycles);  5’-CTTGTTCAGAGC   GGAGAAAGC-  3’(sense)  and  5’-ACATCTGCAAGTACGTTCGTT-3’ (antisense)  for  VEGF  (40  cycles);  and  5’-ACCACAGTCCATGCCATCAC-3’ (sense)  and  5’-TCCACCACCCTGTT GCTGTA-3’ (antisense) for GAPDH (25 cycles). GAPDH mRNA was quantified in each sample as an internal control to normalize the level of mRNA among samples. The PCR products were examined by 2% agarose gel electrophoresis. Data are representative of at least three independent experiments. The relative density of PCR bands were quantified and normalized relative to the control band with the National Institutes of Health (NIH) Image program (Image-J 1.35d, NIH, Bethesda, MD, USA).

### Western blot analysis

Protein was extracted by homogenization of ovaries in the presence of ice-cold lysis buffer (50 mM Tris–HCl (pH 7.5), 150 mM NaCl, 1% Nonidet P-40, and 1 mM EDTA) containing protease inhibitor. The protein content of the cell lysate was determined with Bradford reagent (Bio-Rad, Hercules, CA, USA) using bovine serum albumin (BSA) as the standard. Sixty micrograms of protein were separated by sodium dodecyl sulfate polyacrylamide gel electrophoresis (SDS-PAGE) and transferred to a polyvinylidene fluoride (PVDF) membrane (Millipore, Bedford, MA, USA). The membrane was incubated with anti-Id-1 polyclonal antibody (1:200; Santa Cruz Biotech, Santa Cruz, CA, USA), anti-VEGF monoclonal antibody (1:100; R&D Systems) and anti-β-actin monoclonal antibody (1:5,000; Sigma) in tris-buffered saline (TBS) containing 1% tween 20 (TBS-T) supplemented with skim milk overnight at 4°C. After washing three times with TBS-T, the blotted membranes were incubated with horseradish peroxidase (HRP)-conjugated goat antibody (Santa Cruz Biotech) for 30 minutes at room temperature. After washing three times with TBS-T, the proteins bands were visualized using an enhanced chemiluminescence (ECL) detection system according to the recommended procedure (Amersham Pharmacia Biotech, Piscataway, NJ, USA). Actin expression was used as the control. Data are representative of at least three independent experiments. The relative density of protein bands were quantified and normalized relative to the control band with the National Institutes of Health (NIH) Image program (Image-J 1.35d).

### Immunohistochemistry

Immunohistochemistry was performed on 4 μm-thick, formalin-fixed paraffin sections of ovary tissues using Zymed's SuperPicTure™ Polymer detection system (Zymed Laboratories-Invitrogen, San Francisco, CA, USA). Serial sections of the ovary were mounted on coated slides and placed in an oven at 60°C for 1 hour. The slides were then deparaffinized in xylene and dehydrated in a graded series of ethanol. The slides were boiled in 10 mM citrate buffer (pH 6.0) for 15 minutes in a microwave oven. The endogenous peroxidase was quenched with 0.3% hydrogen peroxide at room temperature for 10 minutes, and then tissues were rinsed four times for 5 minutes each in PBS. The sections were incubated overnight at 4°C with the primary Id-1 antibody (Santa Cruz Biotech) and VEGF antibody (Lab Vision, Fremont, CA, USA) at 1:100 dilutions. The remaining steps were performed according to the instructions supplied with the kit. After washing three times with PBS, the samples were incubated with biotinylated-conjugated secondary antibody and HRP coupled to streptavidin-conjugated antibody for 15 minutes at room temperature and washed three times with PBS. The sections were stained with 3,3-diaminobenzidine (DAB), counterstained with Mayer’s hematoxylin (Sigma), and mounted with histomount solution (Invitrogen). The results were assessed by two pathologists using a light microscope.

### Statistical analysis

An SPSS program (version 12.0) was used for statistical analysis, and all data were presented as a mean±SD. The number of zygotes retrieved and blastocyst formation rate according to treated BMP-6 concentration were analyzed by one-way analysis of variance with post hoc multiple comparisons by Bonferroni-Dunn analysis. Statistical analysis for comparison of expression of Id-1 and VEGF was performed by one-way ANOVA. A *P* value of < .05 was considered statistically significant.

## Results

To investigate whether BMP-6 effects on ovarian response and developmental competency of oocytes in aged female mice, BMP-6 was co-administered to female mice of 26–31 weeks old at different doses (0, 0.01, 0.1, 1, and 10 ng) during superovulation. The mean number of zygotes retrieved was 14.7 in 0.1 ng BMP-6 concentration, which was significantly increased compared to 11.6 in the aged control mice (*P <* 0.05), and this increased number was comparable to 15.1 in the young mice used as a positive control. Especially, 10 ng BMP-6 greatly reduced the number of zygotes retrieved. On the contrary, the mean percentage of fragmented zygotes among those retrieved was 5.3% in 0.1 ng BMP-6, which was significantly lower than those of aged control mice, 0.01 ng and 10 ng BMP-6 concentrations. The development rates to blastocyst stage were also significantly increased in 0.01 ng (28.1%) and 0.1 ng (29.6%) BMP-6 concentrations (*P <* 0.05) when compared with 16.0% in the aged control mice, 11.6% in 1 ng BMP-6 and 3.2% in 10 ng BMP-6. This increased embryo development rates in 0.01 ng and 0.1 ng BMP-6 were similar to that (35.8%) of the young control mice (Table 1).

**Table 1:** Effect of BMP6 treatment on the number and embryo development of zygotes retrieved

**Mice age (weeks)**	**BMP conc. (ng)**	**Mice provided**	**Zygotes flushed**	**Zygotes fragmented (%)**	**Zygotes flushed/mouse**	**Zygotes cultured**	**Blastocyst (%)**
*6-11 (young control)*	0 (control)	7	118	12 (10.2%)	15.1 ± 4.7^b^	106	38 (35.8%)^c^
*26-31 (aged control)*	0 (control)	7	93	12 (12.9%)	11.6 ± 4.6	81	13 (16.0%)
	0.01	8	105	16 (15.2%)	11.1 ± 5.5	89	25 (28.1%)^c^
	0.1	11	171	9 (5.3%)^a^	14.7 ± 2.9^b^	162	48 (29.6%)^c^
	1	10	134	13 (9.7%)	12.1 ± 4.5	121	14 (11.6%)
	10	7	93	30 (32.3%)	9 ± 5.6	63	2 (3.2%)

^a^*P*< .01 (vs. aged control, 0.01 ng, and 10 ng), ^b^*P*< .05 (vs. aged control, 0.01 ng, and 10 ng), ^c^*P*< .05 (vs. aged control, 1 ng and 10 ng).

**Figure 1:**
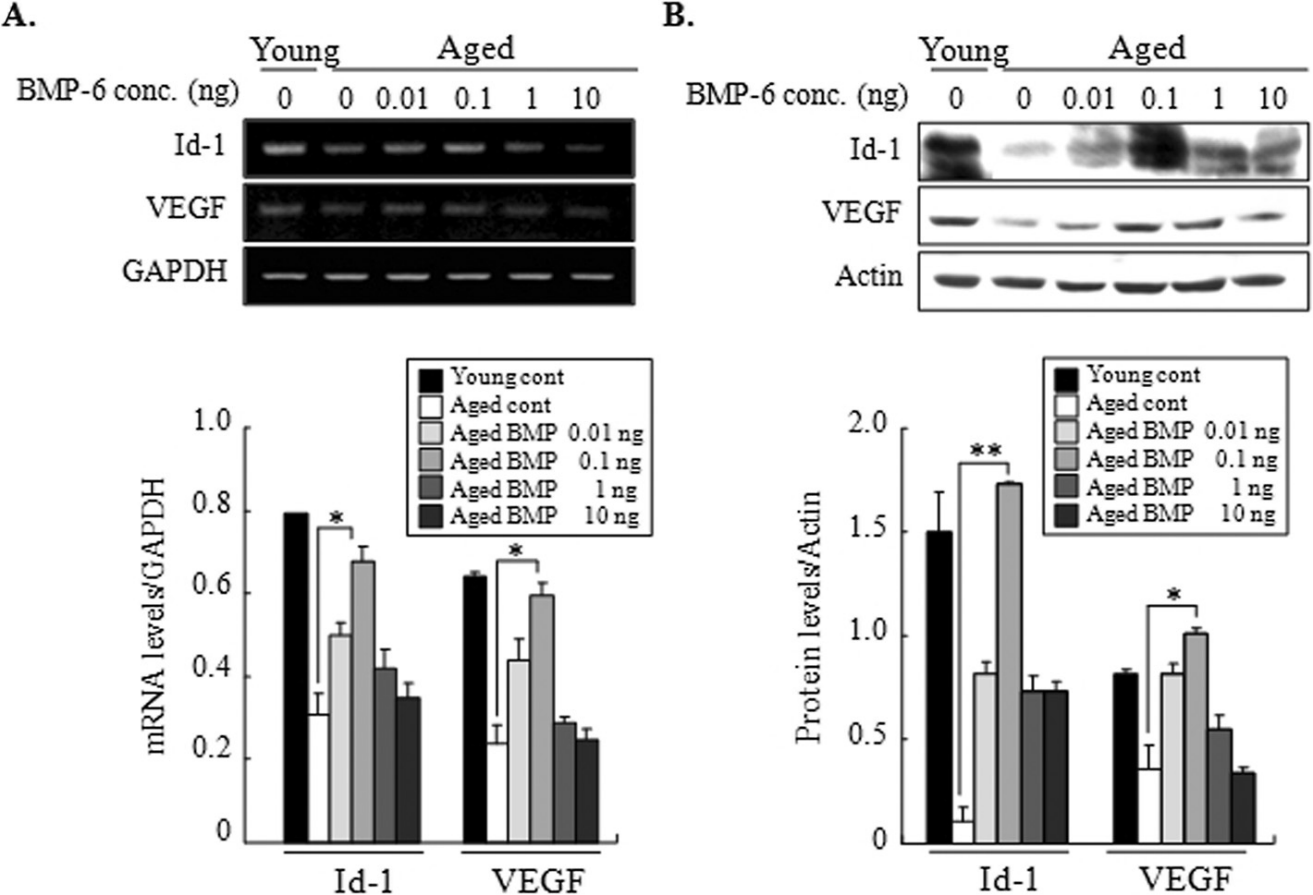
**Effects of administration of BMP-6 on ovarian expressions of Id-1 and VEGF.** Eighteen hours after hCG injection, both whole ovaries of each mouse were collected, and the expressions of ovarian Id-1 and VEGF were examined by RT-PCR and western blot. **(A)** Representative RT-PCR for mRNA expression of ovarian Id-1 and VEGF. Levels of mRNA for Id-1 and VEGF were normalized to the amount of GAPDH per sample. **(B)** Representative western blot products of protein expression for ovarian Id-1 and VEGF. Levels of protein for Id-1 and VEGF were normalized to the amount of actin per sample. Data are representative of at least three independent experiments. The relative density of each gene was quantified with NIH-Image J program (version 1.35d) (^*^P < 0.05 and ^**^P < 0.01).

To investigate whether BMP-6 treatment during superovulation influences ovarian expressions of Id-1 and VEGF, their expressions were examined by RT-PCR and western blot in both ovaries of each mouse just after zygotes collection. Both mRNA and protein expressions of Id-1 and VEGF were significantly stimulated by 0.1 ng BMP-6 compared with aged controls (*P <* 0.05) and the stimulated expression levels were comparable to those of young positive control mice (Figure 1).

**Figure 2:**
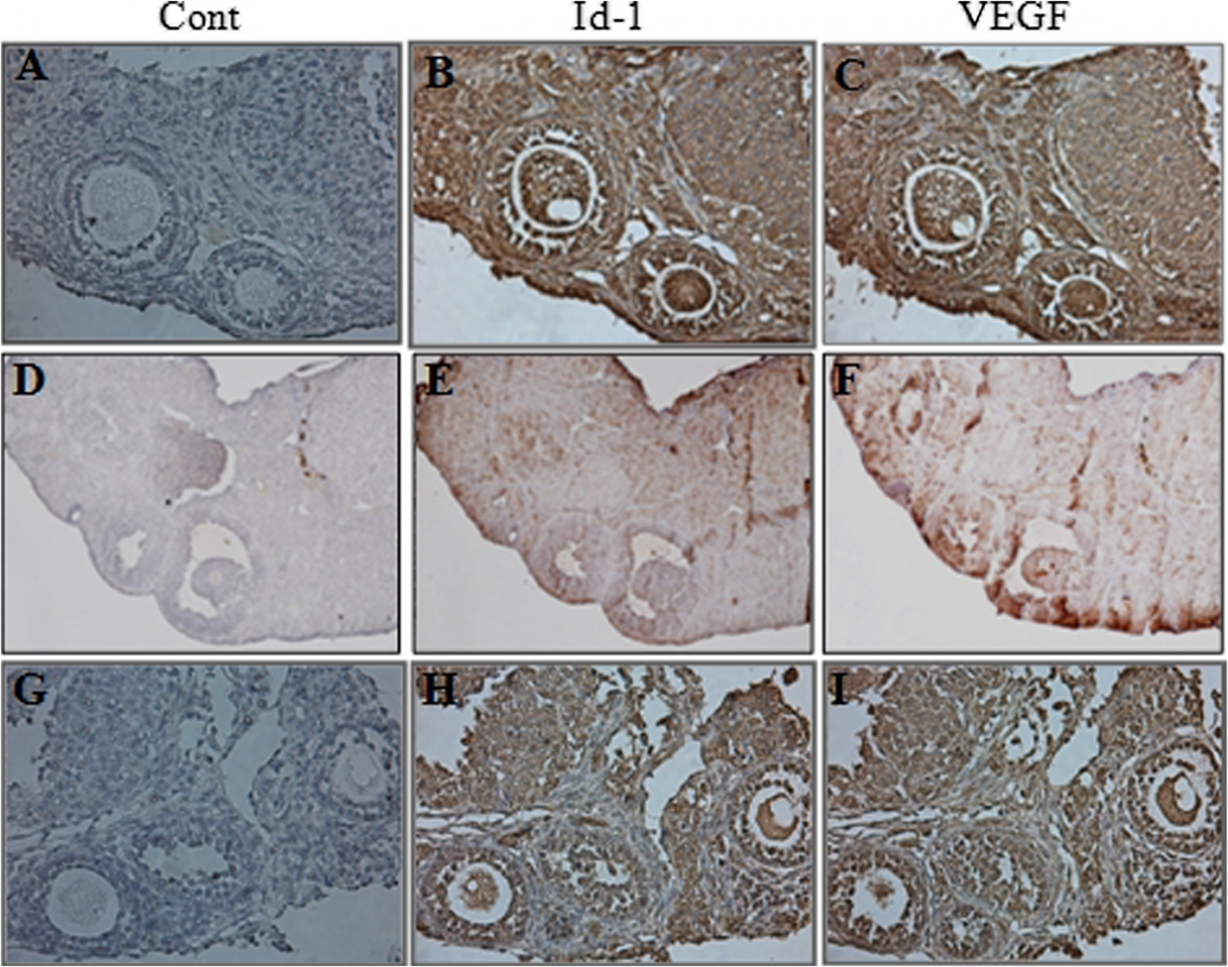
**Immunohistochemical analysis of Id-1 and VEGF in ovaries.** Whole ovaries were collected 18 hours after hCG injection. **(A-C)**, young control mice; **(D-F)**, aged control mice; **(G-I)**, 0.1 ng BMP-6-treated aged mice. Control was immunostained without primary antibody (purple color) **(A, D, G)**. Cell immunostained with Id-1 **(B, E, H)** and VEGF **(C, F, I)**-specific antibody showed a brown color. n =3, six ovaries per aged group (x 100 magnifications).

Immunohistochemistry was performed to evaluate the localized Id-1 and VEGF expression in only 0.1 ng BMP-6-treated ovaries because this dose of BMP-6 resulted in a significantly effect of BMP-6 on ovarian response, oocyte quality, and the expression of Id-1 and VEGF. The expressions of Id-1 and VEGF were localized in oocytes, granulosa cells and stromal cells. The immunoreactivity of Id-1 and VEGF revealed more intense staining compared with aged control mice, but similar intense staining to that of young control (Figure 2).

## Discussion

The present study shows that co-administration of BMP-6 during superovulation in aged female mice improves the number and developmental competence of oocytes ovulated in an appropriate concentration. To our knowledge, this is the first demonstration of the direct effect of BMP-6 on ovarian response, oocyte quality and ovarian expression of angiogenic-related factors of aged female using a mouse model.

The mechanisms for positive effects of BMP-6 on ovarian response and oocyte quality of aged mice are as yet unknown. However, one can consider two aspects; the first is the effect of BMP-6 itself and the second is the indirect effect of BMP-6 via the activation of ovarian angiogenesis.

BMP-6 was abundantly present in the granulosa cells of healthy follicle, not but in atretic follicles, of mammalian ovary [32-34] and it prevents apoptosis of cumulus cells by oocytes in a paracrine manner [17]. These results suggest that BMP-6 may be an important mediator to support healthy follicle growth in follicular development and ovarian function. This hypothesis has been supported by strong evidence of two *in vivo* studies by Campbell *et al*. [21] and Sugiura *et al*. [22]. Campbell *et al.* showed that direct ovarian infusion of BMP-6 resulted in increase in ovarian inhibin A and estradiol secretion [21]. Sugiura *et al*. reported that BMP-6 deficiency in female mice resulted in decreased fertility and less competent to complete preimplantation development [22]. Due to this role of BMP-6, the administration of BMP-6 during superovulation may enhance not only ovarian response but also oocyte quality, subsequently resulting in increased embryo developmental competency.

An interesting finding in the present study was that BMP-6 treatment also stimulated the expression of Id-1 and VEGF in the ovary. This result is consistent with the report by Hogg *et al*. who showed that Id-1 expression was significantly increased following BMP-6 stimulation of granulosa cells *in vitro*[25]. VEGF is a well known representative angiogenic factor and it plays a critical role in the cyclic growth of ovarian follicles and mediates ovarian angiogenesis [3,35].

Id proteins affect endothelial cells proliferation, migration, invasion, and differentiation and have an essential role in angiogenesis [24,30]. Many studies demonstrated that Id-1 stimulates angiogenesis through the activation of VEGF [31,36]. BMP-6 results in an advance in the time of the LH surge [21] and LH surge have some direct effects on angiogenesis to induce a series of cellular and biochemical processes that culminate in ovulation [6]. In this regard, it can be postulated that the beneficial effect of BMP-6 on oocyte quality and ovarian response may be resulted from the activation of ovarian angiogenesis via up-regulation of ovarian Id-1 and VEGF expression. Our previous studies have demonstrated that the activation of ovarian angiogenesis by administration of angiogenic-stimulating factors during ovulation induction could improve ovarian response and oocyte quality with increased expression of ovarian VEGF in aged female mice [37-39]. However, we did not assess ovarian Id-1 and VEGF expression according to the time period after BMP-6 injection during the follicular development. Thus, further study is needed to elucidate when Id-1 and VEGF expression starts and continues after BMP-6 treatment.

Little data is available to confirm physiological concentrations of BMP-6 in the ovary or serum [40]. Furthermore, reports on BMP-6 levels in mice are very limited. Therefore, it was very difficult for us to determine an optimal treatment concentration of BMP-6. Brankin *et al*. investigated the effect of BMP-6 on steroidogenesis and cell proliferation of *in vitro* cultured porcine theca cells by adding various doses (0, 3, 30, 100 ng/mL) of BMP-6 to culture medium [41]. Estradiol production and cell proliferation were increased in 30 ng/ml BMP-6 concentration compared to other concentrations. Shi *et al*. treated human granulosa cells with 100 ng/mL BMP-6 for 24 hours and investigated on gene expression of FSH receptor, inhibin/activin, and anti-Mullerian hormones [15]. Campbell *et al*. directly infused 2 μg of BMP-6 to autotransplanted ovary in sheep and examined ovarian hormone secretion [21]. Considering the BMP concentrations of these studies, we treated 100 ng BMP-6, but the dose severely inhibited the ovarian response and the embryo development rate of retrieved zygotes. Therefore, we serially diluted this dose by ten times and treated with BMP-6 concentrations ranging from 0.01 ng to 10 ng. The treatment of 0.1 ng BMP-6 significantly increased all parameters of the number of zygotes retrieved, blastocyst formation rate, and ovarian Id-1 and VEGF expressions compared to the control and other concentrations, whereas 1 ng and 10 ng BMP-6 decreased the embryo development rate. Especially, 10 ng BMP-6 greatly reduced the number of zygotes retrieved. In this respect, it is thought that 0.1 ng of BMP-6 may be the optimal concentration required to improve ovarian response and oocyte quality during ovarian hyperstimulation in aged mice. The reason for inhibitory effects by high concentrations of BMP-6 (10 ng) on the number of ovulated oocytes and blastoocyst formation is not clear, but may be attributable to the increased apoptosis of granulosa cells [42].

## Conclusions

The present study showed that co-administration of BMP-6 with gonadotropin during superovulation in aged mice increased the ovarian response, developmental competence of oocytes and ovarian Id-1 and VEGF expression. This study provides a notable finding that BMP-6 can stimulate oocyte quality in aged female although this study is done for a mouse model. This result suggests the possibility that this research may have potential clinical implications in the treatment of age-related decline of fertility of women. However, our data are insufficient to apply in the human being. Therefore, first of all to apply the result of the present study, further studies are needed to investigate BMP-6 levels in human follicular fluid, serum, and ovarian tissues according to women’s age, the number and quality of oocytes retrieved during *in vitro* fertilization and embryo transfer.

## Competing interests

The authors declare that they have no competing interests.

## Authors’ contributions

SSP: conception and design, acquisition or analysis of data, and manuscript drafting, made the conception, participated in the design of the study and performed the statistical analysis and manuscript drafting. MJP: conception, design and execution of study, acquisition of data, participated in the conception and design of the study and performed the execution of study and acquisition of data. BSJ: execution of study, analysis of data and manuscript drafting, performed the execution of study, analysis of data and manuscript drafting. JKJ: analysis and interpretation of data, participated in analysis and interpretation of data. JBS: analysis and critical discussion, participated in analysis and critical discussion. KSL: drafting the manuscript or revising it critically for important intellectual content, and final approval of the version to be published, participated in drafting the manuscript or revising it critically for important intellectual content, and final approval of the version to be published. All authors read and approved the final manuscript.
